# Extensive Crosstalk between *O*-GlcNAcylation and Phosphorylation Regulates Akt Signaling

**DOI:** 10.1371/journal.pone.0037427

**Published:** 2012-05-22

**Authors:** Shuai Wang, Xun Huang, Danni Sun, Xianliang Xin, Qiuming Pan, Shuying Peng, Zhongjie Liang, Cheng Luo, Yiming Yang, Hualiang Jiang, Min Huang, Wengang Chai, Jian Ding, Meiyu Geng

**Affiliations:** 1 Division of Antitumor Pharmacology, Shanghai Institute of Materia Medica, Chinese Academy of Sciences, Shanghai, China; 2 Laboratory of Mass Spectrometry, Shanghai Institute of Materia Medica, Chinese Academy of Sciences, Shanghai, China; 3 Center for Drug Discovery and Design, State Key Laboratory of Drug Research, Shanghai Institute of Materia Medica, Chinese Academy of Sciences, Shanghai, China; 4 Glycosciences Laboratory, Faculty of Medicine, Imperial College London, Harrow, United Kingdom; University of Nebraska Medical Center, United States of America

## Abstract

*O*-linked N-acetylglucosamine glycosylations (*O*-GlcNAc) and *O*-linked phosphorylations (*O*-phosphate), as two important types of post-translational modifications, often occur on the same protein and bear a reciprocal relationship. In addition to the well documented phosphorylations that control Akt activity, Akt also undergoes *O*-GlcNAcylation, but the interplay between these two modifications and the biological significance remain unclear, largely due to the technique challenges. Here, we applied a two-step analytic approach composed of the *O*-GlcNAc immunoenrichment and subsequent *O*-phosphate immunodetection. Such an easy method enabled us to visualize endogenous glycosylated and phosphorylated Akt subpopulations in parallel and observed the inhibitory effect of Akt *O*-GlcNAcylations on its phosphorylation. Further studies utilizing mass spectrometry and mutagenesis approaches showed that *O*-GlcNAcylations at Thr 305 and Thr 312 inhibited Akt phosphorylation at Thr 308 via disrupting the interaction between Akt and PDK1. The impaired Akt activation in turn resulted in the compromised biological functions of Akt, as evidenced by suppressed cell proliferation and migration capabilities. Together, this study revealed an extensive crosstalk between *O*-GlcNAcylations and phosphorylations of Akt and demonstrated *O*-GlcNAcylation as a new regulatory modification for Akt signaling.

## Introduction

The serine/threonine kinase Akt, also known as protein kinase B (PKB), is a central node in cell signaling downstream of growth factors, and regulates diverse cellular biological functions including cell proliferation, survival, and metabolism. Aberrant loss or gain of Akt activation underlies a variety of human diseases, including diabetes and cancer [1]–[4]. Because of the important roles of Akt signaling in cancer, this pathway has been an attractive target for the development of novel anticancer drugs. However, as Akt signal is also important for normal cell functions, targeting Akt is likely to raise serious side effect. Thus, the comprehensive understanding of the regulatory mechanism of Akt signaling will be of importance to design better therapeutic strategies [3], [5].

Posttranslational modifications (PTM) often play important roles in regulating protein functions [6], [7]. In response to growth factor stimulation, Akt is recruited to the plasma membrane, and phosphorylated at Thr 308 by the phosphoinositide-dependent kinase 1 (PDK1) [8], [9] and Ser 473 by the mammalian target of rapamycin complex 2 (mTORC2) [8], [10], respectively. The two phosphorylations are required for the full activation of Akt [2]. Conversely, the dephosphorylation leads to the termination of Akt activity. The Thr308 phosphorylation is dephosphorylated by the protein phosphatase 2A (PP2A) [11], [12], and the Ser473 phosphorylation is removed by the PH domain leucine-rich repeat protein phosphatase (PHLPP) [13]. To date, the phosphorylation and dephosphorylation mechanism has been thought to primarily control Akt signaling, but other posttranslational modifications associated with Akt activity remain poorly known. Until recently, two new modifications of Akt, monoubiquitination and polyubiquitination, were reported, which were related to the activation and degradation of Akt, respectively [14], [15].

Protein *O*-GlcNAcylation is a dynamic posttranslational modification at serine or threonine residues of nuclear and cytoplasmic proteins, and competes with phosphorylation at the same or proximal sites. The interplay between *O*-GlcNAc and *O*-phosphate has been implicated in regulating protein function and plays an important role in the aetiology of chronic diseases, such as diabetes and cancer [16]–[18]. In addition to phosphorylations, Akt also undergoes *O*-GlcNAc modification [19]. Since its discovery almost one decade ago, Akt has become one of the most frequently investigated *O*-GlcNAcylated target proteins. In spite of the extensive studies, the relationship between *O*-GlcNAcylations and phosphorylations of Akt appears controversial [19]–[26]. For instance, in 3T3-L1 adipocytes, *O*-GlcNAcase inhibitor PUGNAc treatment suppressed the phosphorylation at Thr 308 [19], [20], which, however, failed to be recapitulated by other two selective *O*-GlcNAcase inhibitor, NButGT and 6-Ac-Cas [21], [22]. These discrepancies indicate that caution should be taken when cells are treated with *O*-GlcNAcase inhibitors or subjected to OGT overexpression, because ambiguous results might be obtained and may complicate analyses of the crosstalk between *O*-GlcNAcylation and phosphorylation. [27]–[31].

A recent report described a mass-tagging strategy using polyethylene glycol tag for *O*-GlcNAc study, and showed that the difference of the phosphorylation level between the glycosylated and non-glycosylated forms of a given protein could reflect the impact of *O*-GlcNAcylation on phosphorylation [32]. Based on this conceptual breakthrough, we designed a new antibody-based approach to directly investigate this crosstalk independent of the chemical labels. Using this method in combination with mass spectrometry and Tyr residue simulated *O*-GlcNAc modifications, we revealed an extensive and complex crosstalk between *O*-GlcNAcylations and phosphorylations of Akt, showing that *O*-GlcNAcylations of Akt at Thr 305 and 312 inhibited its phosphorylation at Thr 308 by disrupting the interaction between Akt and PDK1. Our data suggest that *O*-GlcNAcylation represents a new regulator for Akt.

## Results

### Akt *O*-GlcNAcylations Inhibit its Phosphorylations

**Figure 1:**
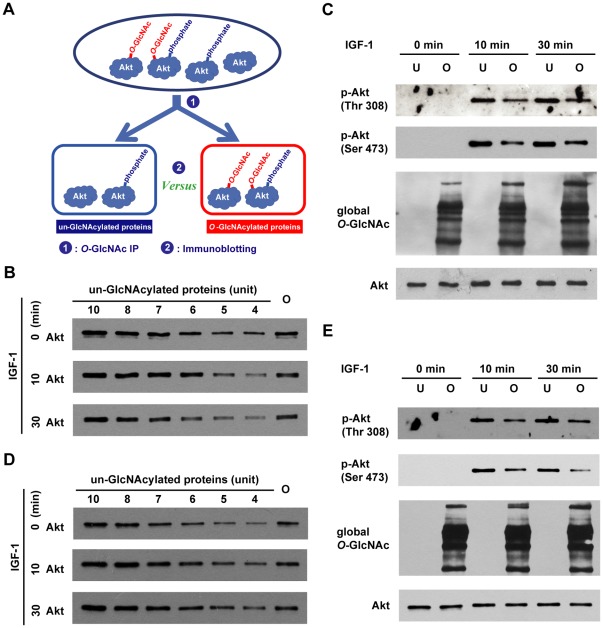
*O*-GlcNAcylations of endogenous Akt inhibit Akt phosphorylations at Thr 308 and Ser 473. (**A**) Schematic representation of the method for characterization of the interplay between *O*-GlcNAc and *O*-phosphate of Akt. The proteins in the cell are first divided into two pools by *O*-GlcNAc immunoprecipitation: the un-GlcNAcylated and *O*-GlcNAcylated proteins subpopulations. Next, the phosphorylation levels of Akt between the two subpopulations are measured by western blotting. This will directly reflect the effect of *O*-GlcNAcylations on the phosphorylations of Akt. Here, MCF-7 cells were serum-starved under PUGNAc treatment (**B** and **C**) or under normal conditions (**D** and **E**), followed by IGF-1 stimulation for the indicated times. The un-GlcNAcylated and *O*-GlcNAcylated proteins subpopulations were prepared. Akt *O*-GlcNAcylations under PUGNAc treatment: (**B**) Determination of the same level of total Akt between the *O*-GlcNAcylated and un-GlcNAcylated proteins subpopulations. The *O*-GlcNAcylated proteins subpopulation was compared to a series of known amounts of the un-GlcNAcylated proteins subpopulations by immunoblotting against total Akt. (**C**) Comparison of the phosphorylation levels of the un-GlcNAcylated and *O*-GlcNAcylated Akt. The un-GlcNAcylated and *O*-GlcNAcylated proteins subpopulations were immunoblotted against Akt, *O*-GlcNAc, and the phosphorylations at Thr 308 and Ser 473. The constitutive *O*-GlcNAcylations of Akt: (**D**) Determination of the same level of total Akt between the *O*-GlcNAcylated and un-GlcNAcylated proteins subpopulations. The *O*-GlcNAcylated proteins subpopulation was compared to a series of known amounts of the un-GlcNAcylated proteins subpopulations by immunoblotting against total Akt. (**E**) Comparison of the phosphorylation levels of the un-GlcNAcylated and *O*-GlcNAcylated Akt. The un-GlcNAcylated and *O*-GlcNAcylated proteins subpopulations were subjected to immunoblot assay of Akt, *O*-GlcNAc, and the phosphorylations at Thr 308 and Ser 473. One unit was equal to 0.01 µl of the un-GlcNAcylated proteins. U: un-GlcNAcylated proteins; O: *O*-GlcNAcylated proteins.

To investigate the interplay between *O*-GlcNAc and *O*-phosphate modifications of Akt, we applied a two-step analytic approach (**Fig. 1A**), which allowed us to directly visualize the endogenous glycosylated Akt and assess its phosphorylation. The experimental strategy was to fractionate the glycosylated and nonglycosylated Akt using *O*-GlcNAc immunoenrichment, followed by the immunodetection of phosphorylation using site-specific antibodies.

Briefly, MCF-7 cells were treated with *O*-GlcNAcase inhibitor PUGNAc to facilitate the *O*-GlcNAcylated proteins enrichment. A pan-*O*-GlcNAc antibody was applied for *O*-GlcNAc immunoprecipitation which yielded the *O*-GlcNAcylated fraction in precipitate and the supernatant being the un-GlcNAcylated subpopulations. Then, the global protein amount of the two fractions were adjusted using a two-step quantitative immunoblotting analysis (see details in Materials and Methods) to narrow down the amount of the *O*-GlcNAcylated Akt to the linear range of immunoblotting analysis (**Fig. 1B**), and to ensure the identical total Akt amount between the *O*-GlcNAcylated and non-GlcNAcylated subpopulations. The confirmed Akt level and clean fractionation of *O*-GlcNAcylated subpopulations (**Fig. 1C**) markedly enhanced the credibility of the phosphorylation assessment afterwards. A simple inspection of the blotting clearly showed that the phosphorylation levels at Thr 308 and Ser 473 of *O*-GlcNAcylated Akt were considerably less than those of un-GlcNAcylated Akt (**Fig. 1C**), suggesting that the PUGNAc-induced Akt *O*-GlcNAc modifications suppressed its phosphorylations at both Thr 308 and Ser 473. This results were recapitulated by detecting the constitutively *O*-GlcNAcylated Akt, which importantly excluded the potential interference resulted from *O*-GlcNAcase inhibitors treatment (**Fig. 1D and E**). 

It was noticed that *O*-GlcNAcylated Akt underwent slight phosphorylation at Thr 308 and Ser 473 (**Fig. 1C and E**). While we could not rule out the possibility of contamination between the subpopulations samples, it was more likely that Akt bore multiple *O*-GlcNAcylation sites and some of them inhibited Akt phosphorylations.

### Akt is *O*-GlcNAcylated at Thr 305, Thr 312, Ser 126 and Ser 129

**Figure 2:**
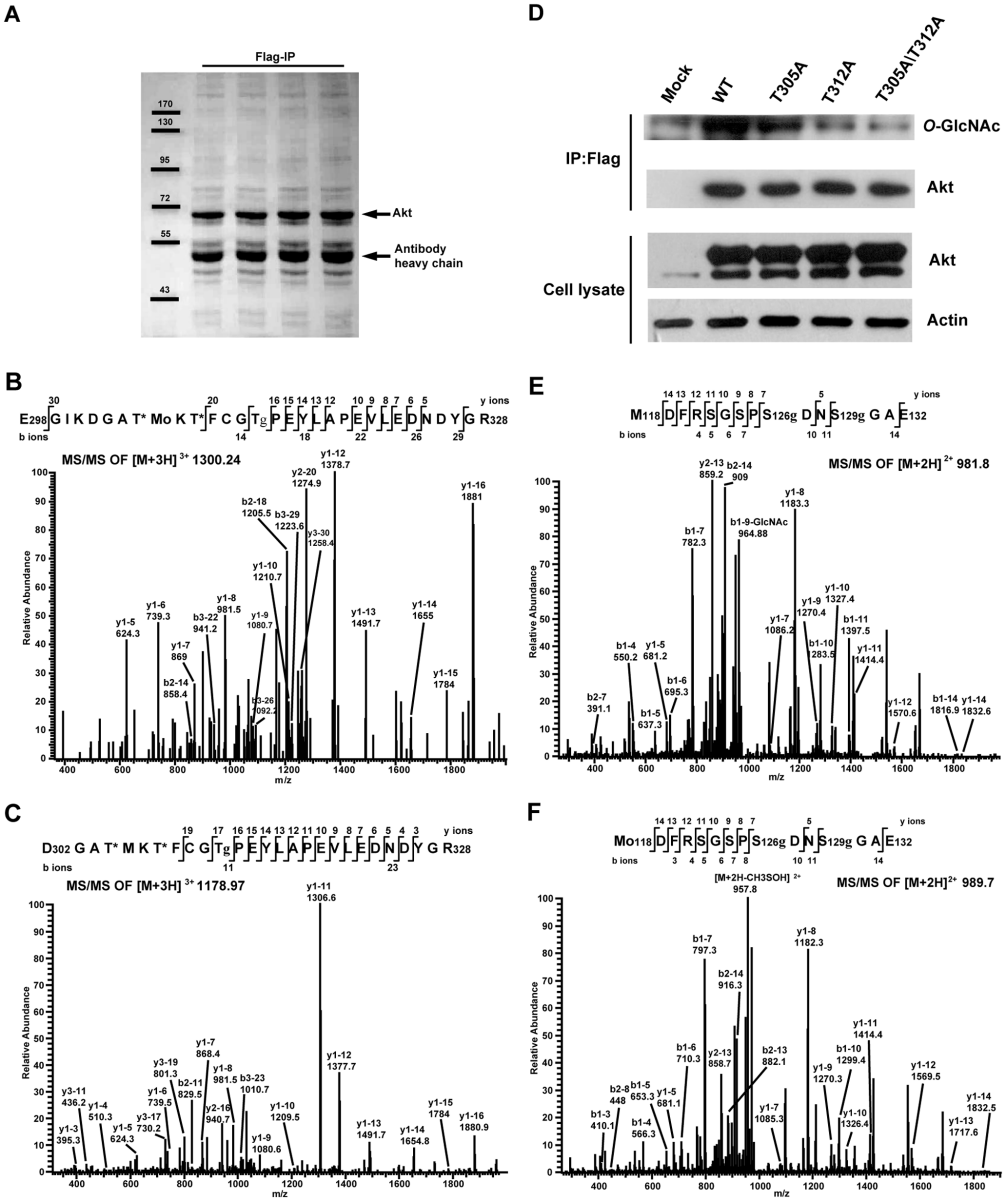
Identification of Akt *O*-GlcNAcylation sites at Thr 305, Thr 312, Ser 126 and Ser129. (**A**) Exogenous Akt was immunoprecipitated from MCF-7 cells under PUGNAc treatment. The eluted proteins were further separated by SDS-PAGE. Akt bands were in-gel digested and analyzed by mass spectrometry. (**B**) Tandem mass spectrum of the triply charged precursor ion at m/z 1300.24 with *Xcorr* score 2.87. The spectrum corresponded to Akt peptide 298–328 bearing one oxidation at Met 306 and two *O*-GlcNAc moieties. The two possible *O*-GlcNAcylation sites were Thr 312 and one of Thr 305 and 308. (**C**) Tandem mass spectrum of the triply charged precursor ion at m/z 1178.97 with *Xcorr* score 3.29. This spectrum corresponded to Akt peptide 302–328 containing one *O*-phosphate and two *O*-GlcNAc moieties. The two potential *O*-GlcNAcylation sites were Thr 312 and one of Thr 305 and 308. (**D**) *O*-GlcNAc levels of wild type and mutants of Akt. Akt and its mutants were immunoprecipitated from MCF-7 cells, and then subjected to western blotting analysis of total Akt and *O*-GlcNAc level. (**E**) Tandem mass spectrum of the doubly charged precursor ion at m/z 981.8 with *Xcorr* score 2.64. The spectrum corresponded to Akt peptide 118–132 bearing two *O*-GlcNAc modifications at Ser 126 and Ser 129. (**F**) Tandem mass spectrum of the doubly charged precursor ion at m/z 989.7 with *Xcorr* score 2.70. This spectrum corresponded to Akt peptide 118–132 bearing one oxidation at Met 118 and two *O*-GlcNAc modifications at Ser 126 and Ser 129. g: *O*-GlcNAc; o: oxidation, *: possible site for *O*-GlcNAcylation or phosphorylation.

To determine which sites of *O*-GlcNAc modifications inhibited Akt phosphorylations, we identified Akt *O*-GlcNAcylation sites by tandem mass spectrometry. Ectopically expressed Akt was immunoprecipitated from MCF-7 cells treated by *O*-GlcNAcase inhibitor PUGNAc and further purified by SDS-PAGE (**Fig. 2A**). Akt bands were in-gel digested with trypsin or Glu-C and the peptides were analyzed by mass spectrometry [33]. With the sequence coverage of 85% of human AKT1, we identified four different *O*-GlcNAc-modified peptides revealing four *O*-GlcNAcylation sites.

The two different modified forms of peptides from the activation loop were identified. **Fig. 2B** showed one tandem mass spectrum corresponding to Akt peptide 298–328 containing two *O*-GlcNAc residues. In this spectrum, the C-terminal residues 314–328 were confidently assigned by the presence of a continuous series of unmodified y-ions (y5–y10 and y12–y16), suggesting that the two *O*-GlcNAcylation sites occurred at the N-terminal part. Furthermore, Thr 312 was deduced as one *O*-GlcNAcylation site by the presence of a modified doubly charged fragment ions y2–20. **Fig. 2C** showed another tandem mass spectrum corresponding to Akt peptide 302–328 bearing one phosphate and two *O*-GlcNAc moieties. Similarly, the presence of a continuous series of unmodified y-ions (y3–y16) confirmed the identity of the C-terminal part of peptide 314–328, thereby narrowing down the three modifications to the N-terminal part. Again, the *O*-GlcNAcylation site at Thr 312 was deduced by the presence of the modified fragment ions y3–17 (triply charged) and y3–19 (triply charged). Considering that Thr 308 was a known phosphorylation site, our results suggested that Thr 305 and 312 were likely two *O*-GlcNAcylation sites. To verify further the identified *O*-GlcNAc sites, we generated site-specific mutants of Akt at Thr 305 and Thr 312, and examined their *O*-GlcNAc levels. Less *O*-GlcNAc levels were detected on both of single mutants, particularly T312A, compared to wild type (**Fig. 2D**). Moreover, double mutant of T305A/T312A showed much less *O*-GlcNAc level (**Fig. 2D**). This confirmed *O*-GlcNAcylation sites of Akt at Thr 305 and Thr312.

We also identified other two *O*-GlcNAcylated peptides 118–132 containing two *O*-GlcNAc residues with or without Met oxidation, consistently revealing Ser 126 and Ser 129 as two *O*-GlcNAc modification sites (**Fig. 2E and F**). Given that Ser 126 and 129 are also known phosphorylation sites [34], [35], the simple site-directed mutagenesis will be unable to distinguish the biological functions mediated by either *O*-GlcNAcylations or phosphorylations [16]. Together, we have determined four *O*-GlcNAcylation sites of Akt: Thr 305 and 312 in the activation loop, and Ser 126 and 129 at the known phosphorylation sites. Among the four *O*-GlcNAcylation sites, Thr 305 and 312 were particularly intriguing to us, considering that the endogenous *O*-GlcNAc-modified Akt exhibited the reduced phosphorylation level at the proximal Thr 308 (**Fig. 1C and E**).

### *O*-GlcNAcylations at Thr 305/312 Suppress Akt Phosphorylation at Thr 308 via Disrupting its Interaction with PDK1

**Figure 3:**
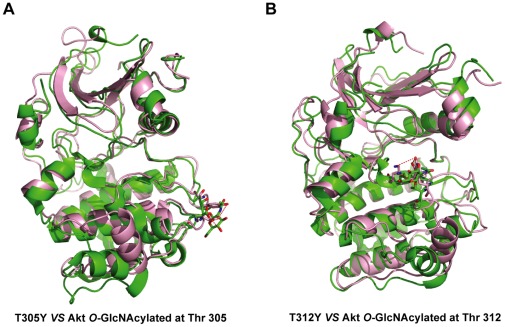
Molecular modeling analysis. (**A**) Molecular modeling of T305Y mutant (pink) and Akt *O*-GlcNAcylated at Thr 305 (green). Tyr305, *O*-GlcNAcylated Thr305 and Asp325 are represented as sticks. (**B**) Molecular modeling of T312Y mutant (pink) and Akt *O*-GlcNAcylated at Thr 312 (green). Tyr312, *O*-GlcNAcylated Thr312, Asp274 and Lys276 are shown as sticks. Additionally, oxygen atoms are shown in red, nitrogen atoms are shown in blue, and the hydrogen bonds are shown in red dot lines.

Distinct from other post-modifications like phosphorylation, methylation and acetylation, *O*-GlcNAcylation presents a large volume of modification. The presence of *O*-GlcNAcylations has been known to generate the bulky steric hindrance and result in the alteration of local conformation. We reasoned that substitution of *O*-GlcNAc-modified Thr residue with amino acids with the relatively large volume of side chain, e.g. Tyr, would likely simulate the inhibitory effect of *O*-GlcNAcylation. To test the possibility of this hypothesis, molecular dynamics (MD) simulations were performed on the two *O*-GlcNAc modification sites at Thr 305 and 312 substituted by *O*-GlcNAc or Tyr residue, based on the X-ray crystal structure of AKT1 [36]. For *O*-GlcNAc modifications, the MD simulations suggested that the interactions between the phosphothreonine and His194 in the αC helix, Arg 273 in the catalytic loop and Lys 297 in the activation loop (A loop) were not preserved any more in both *O*-GlcNAc-modified Akt, as compared to wild-type Akt. Meanwhile, the αC helix rotated outward and the A loop refolded in the *O*-GlcNAc modifications, partially similar to the dephosphorylated conformation (**Fig.3A and B** in green). For Tyr substitutions, the modeling results indicated that Tyr 305 H-bonded with Asp325, and Tyr312 H-bonded with Asp 274 and Lys 276, similar to the *O*-GlcNAc modifications (**Fig. 3A and B** in pink). Therefore, these results indicated that it was possible that the local conformational alterations caused by Tyr substitutions were similar to that caused by *O*-GlcNAc modifications and the steric hindrance effect of *O*-GlcNAcylations at Thr 305 and 312 could, to some extent, be mimicked by Tyr residue.

**Figure 4:**
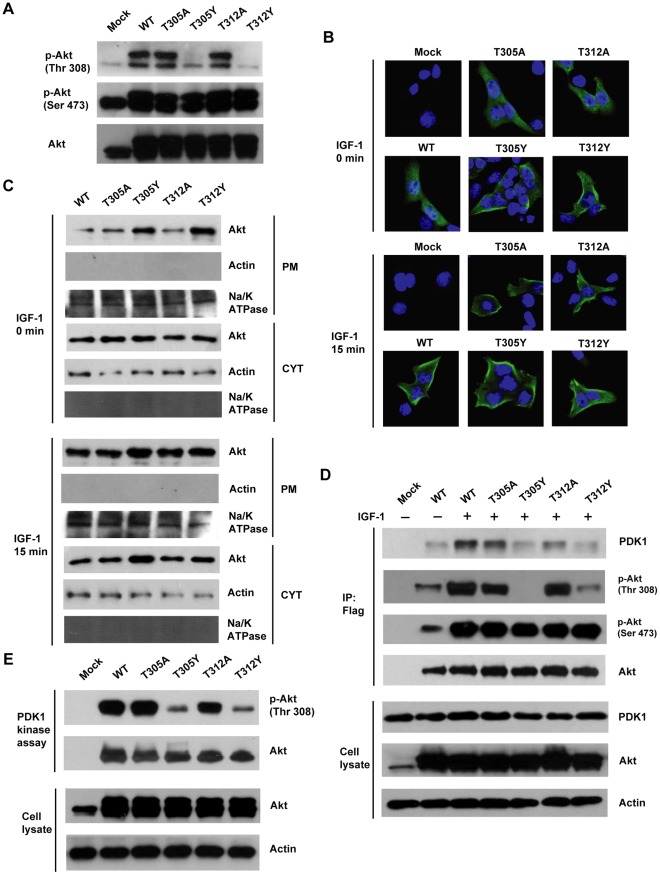
*O*-GlcNAclyations at Thr 305/312 suppress the Thr308 phosphorylation via disrupting the interaction between Akt and PDK1. (**A**) Immunoblot analysis of the phosphorylation levels of wild-type Akt and its mutants. MCF-7 cells were transfected by the indicated plasmids, followed by IGF-1 stimulation. The cell lysates were subjected to immunoblotting analysis of the phosphorylation levels of Akt. (**B**) Fluorescence images of the subcellular localization of Akt and its mutants in COS-7 cells. COS-7 cells were transfected by the indicated plasmids and serum-starved overnight, followed by IGF-1 stimulation. Cells were fixed and probed by primary Akt antibody. (**C**) Immunoblot analysis of subcellular fractions of COS-7 cells expressing wild-type Akt and its mutants in response to IGF-1 stimulation. Na/K ATPase and actin were used as the plasma membrane and cytoplasmic markers, respectively. PM, plasma membrane; CYT, cytosol. (**D**) Coimmunoprecipitation assay for the interaction between PDK1 and Akt or its mutants. MCF-7 cells were transfected by the indicated plasmids and treated by IGF-1. Akt and its mutants were immunoprecipitated with anti-Flag agarose and the precipitate were probed against Akt and PDK1. (**E**) *In vitro* Kinase assay of PDK1. Akt and its mutants were immunoprecipitated from overexpressed MCF-7 cells and incubated with PDK1 at room temperature. The reaction mixture were immunoblotted against the Thr308 phosphorylation and total Akt.

We next examined whether T305Y and T312Y mutants could exhibit the similar reduced phosphorylation level at Thr 308 as the endogenous *O*-GlcNAcylated Akt (**Fig. 1C and E**). We observed that T305Y and T312Y mutants showed the same expression level as wild type (**Fig. 4A**). In agreement with the inhibitory effect of *O*-GlcNAcylations of endogenous Akt on the Thr308 phosphorylation (**Fig. 1C and E**), Tyr mutations at both Thr 305 and Thr 312 almost completely abolished the phosphorylation at Thr 308 stimulated by IGF-1, but no effect was detected for the phosphorylation at Ser 473, as compared to wild type and T305A and T312A mutants (**Fig. 4** **A**). Plus, *O*-GlcNAcylation or phosphorylation did not occur on Tyr305 or Tyr312 (data not shown). Thus, these cellular results indicated that Tyr substitution could simulate the steric hindrance of *O*-GlcNAcylations at Thr 305 and 312 that inhibited the Thr308 phosphorylation of Akt.

The translocation to the plasma membrane, where Akt is phosphorylated at Thr 308 by PDK1, is a key step for Akt activation [8], [9]. We next tested whether *O*-GlcNAcylation affected the plasma membrane localization of Akt. Immunofluorescence staining was applied to examine the subcellular localization of wild-type Akt and the T305 and T312 mutants in COS-7 cells upon IGF-1 stimulation. We observed that all the mutants and wild-type Akt translocated to the plasma membrane within 15 min after IGF-1 stimulation. Indeed, we detected the increased membrane localization of both T305Y and T312Y mutants compared to wild-type Akt and the T305A and T312A mutants (**Fig. 4B and 4C**). Same results were obtained using subcellular fractionation followed by immunoblotting analysis. It was important to mention that the functional translocation of T305Y and T312Y mutants upon IGF induction excluded the possibility that abolished phosphorylation observed above was solely due to the conformational disruption of the mutants. These results together indicated that the *O*-GlcNAcylations caused defect in Akt Thr 308 phosphorylation was unlikely due to the impaired plasma membrane recruitment.

Next we investigated whether *O*-GlcNAcylations at Thr 305 and 312 affected the interaction between PDK1 and Akt. The coimmunoprecipitation results showed that T305Y and T312Y mutants had the considerably decreased interaction with PDK1 compared to wild-type Akt, T305A and T312A mutants (**Fig. 4D**). Importantly, *in vitro* PDK1 enzymatic assay showed that T305Y and T312Y mutants exhibited the significantly reduced phosphorylation level at Thr 308, as compared to wild-type Akt and T305A and T312A mutants (**Fig. 4E**). Thus, these studies suggested that the *O*-GlcNAcylations at Thr 305 and Thr 312 likely blocked the access of PDK1 to Thr 308 of Akt and suppressed the subsequent phosphorylation at Thr 308. Together, these results suggested that *O*-GlcNAc modifications at Thr 305 and 312 were associated with the inhibition of the Thr308 phosphorylation through disrupting the interaction between Akt and PDK1.

### *O-*GlcNAcylations at Thr 305/312 Down-regulates Akt Activity

**Figure 5:**
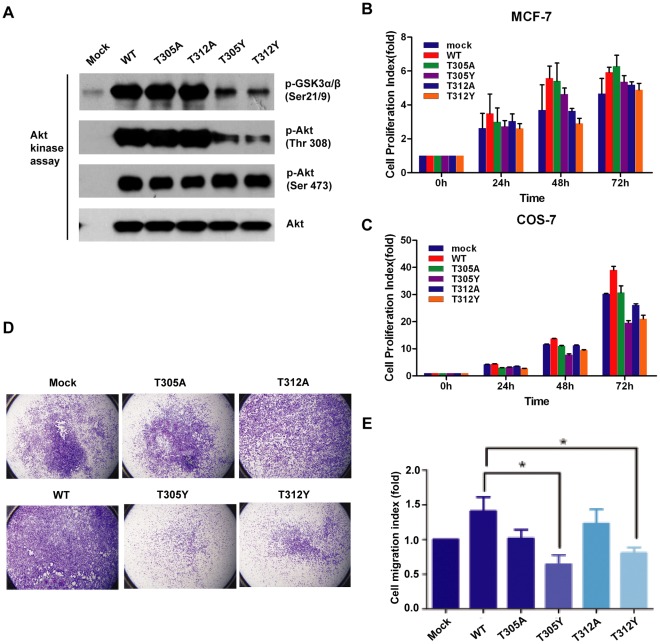
Abnormal *O*-GlcNAcylations down-regulate Akt activity and functions. (**A**) *In vitro* Kinase assay of Akt. MCF-7 cells were transfected by the indicated plasmids and treated by IGF-1. The immunoprecipitated Akt and its mutants were incubated with substrate GSK-3 fusion protein, followed by immunoblotting against the GSK3α/β(Ser21/9) phosphorylation, Akt phosphorylations and total Akt. (**B&C**) Cell proliferation assay of Akt and its mutants. MCF-7 and COS-7 cells transfected by wild-type Akt and its mutants were plated and cultured for the indicated times. Then, the cells were trypsinized and counted relative to the initially seeded cell number. Representative result was shown from three independent experiments with similar tendency. (**D**) Cell migration assay of wild-type Akt and its mutants. COS-7 cells were transfected by wild-type Akt and its mutants, and were tested using Transwell cell migration assay. The migrated cells were stained by crystal violet. (**E**) Quantification analysis of cell migration effect of Akt and its mutants. The stained cells were extracted with a solution of 10% acetic acid, which absorbance at 600 nm was measured. Cell migration levels were presented as mean ± s.d. from three independent experiments.

Given that *O*-GlcNAcylations at Thr 305 and 312 inhibited Akt phosphorylation at Thr 308 which was thought as the most critical phosphorylation for Akt activation [5], we sought to determine the effect of *O*-GlcNAcylations at these two sites on Akt activities and functions. We examined the kinase activity of wild type and the mutants of Akt using an *in vitro* Akt kinase activity kit, where the recombinant GSK3-fusion protein containing residues surrounding GSK-3α/β (Ser21/9) was provided as an Akt substrate. In line with the decrease of Akt Thr308 phosphorylation, T305Y and T312Y mutants exhibited markedly reduced kinase activity compared to wild-type Akt and the T305A and T312A mutants by measuring phospho-GSK3 ratio (**Fig. 5A**). This indicated that *O*-GlcNAcylations at Thr 305 and 312 inhibited Akt kinase activity, thereby in turn likely affecting the biological functions of Akt mediated by the Thr308 phosphorylation.

Akt is one of the most versatile kinases in human kinome and controls an array of diverse cellular functions. Cell proliferation is one of the most prominent biological functions of Akt [2], [3]. Consistent to the previous report [15], introduction of wild-type Akt markedly enhanced the cell proliferation rate of MCF-7 or COS-7 cells. However, T305Y and T312Y mutants showed decreased cell proliferation effect compared to wild-type Akt especially in COS7 cells (**Fig. 5B and 5C** **)**. These observations suggested that the presence of *O*-GlcNAc modifications at Thr 305 and Thr 312 attenuated the Thr308 phosphorylation-mediated cell proliferation effect of Akt. Cell migration and invasion are emerging as an intriguing role of Akt [2]. It has been reported that epidermal growth factor could stimulate the migration of COS-7 cells [37]. Similar to the previous study, we observed that introduction of wild-type Akt markedly enhanced the migration of COS-7 cells, whereas T305Y and T312Y mutants exhibited the evidently reduced cellular migration efficacy, as compared to wild-type Akt (**Fig. 5D and 5E**). Thus, these results indicated that *O*-GlcNAcylations at Thr 305 and Thr 312 affected COS-7 cell migration mediated by the Thr308 phosphorylation of Akt. Furthermore, *O*-GlcNAc-deletion mutants T305A and T312A also showed the moderate reduction of cell proliferation and migration compared to wild type (**Fig. 5B–E**). Thus, it was likely that *O*-GlcNAcylations at Thr 305 and 312 could directly regulate the biological functions of Akt. Together, our results indicated that abnormal *O*-GlcNAc modifications at Thr 305 and Thr 312 could down-regulate the normal activity and functions of Akt.

## Discussion

Although the crosstalk between *O*-GlcNAcylation and phosphorylation is getting increasing attention as an important regulatory mechanism, the progress in understanding this crosstalk has been highly hindered by the technique challenges. Here, we provided a useful approach for decoding this important interplay of an endogenous protein. While our strategy, to some extent, echoed a recent work by Rexach *et al*, which successfully visualized the interplay of a specific protein by introducing a chemical mass tag [32], our method exhibited two evident features. Firstly, it was more simplified by employing only commercially available antibodies and routine laboratory techniques, independent of techniquely-challenging manipulations such as chemical synthesis and mass spectrometry; Secondly, the immuno-enrichment application using an *O*-GlcNAc antibody allowed us to analyze *O*-GlcNAcylated proteins in a very low abundance. This feature became essential in the case of Akt where only a very small portion of Akt got *O*-GlcNAcated, as consistently observed in this study and previous reports [19]. Moreover, it was also deserved to be emphasized that the same level of total Akt between the glycosylated and unglycosylated fractions was determined prior to the comparison of the phosphorylation levels, as it made the accurate and straightforward comparison afterwards become possible. In addition to *O*-GlcNAcylation, crosstalks between different types of PTMs such as methylation, acetylation, ubiquitination and phosphorylation have been widely observed as the regulatory mechanism of PTMs [38]–[40]. We believe our approach provides a simple and effective technique option for understanding these processes by solely applying the different pan- or specific- antibodies.

In this study, we applied a tyrosine residue with large volume of side chain to simulate the bulky steric hindrance of *O*-GlcNAcylation, which enabled us to explore the interplay between the site-specific *O*-GlcNAcylations and phosphorylations. Such a strategy was supported by our molecular modeling analysis (**Fig. 3**), showing that substitution of *O*-GlcNAc-modified Thr residues with Tyr would likely simulate the steric hindrance of *O*-GlcNAcylation, though we have been aware of the structural difference between Tyr and *O*-GlcNAc. It is conceivable that this strategy is particularly useful to study the impact of *O*-GlcNAcylations on other modifications occurring on the adjacent residues. Importantly, we have validated this strategy by testing the two reported *O*-GlcNAcylated proteins, p53 [41] and Snail1 [42]. The Tyr mutants at *O*-GlcNAcylation sites of both p53 and Snail1 showed significantly reduced phosphorylation occurring on adjacent residues, as compared to wild type (**Fig. S1A** and **S1B**). By using T305Y and T312Y mutants of Akt, we observed that the two mutants reduced phosphorylation levels at Akt Thr 308, consistent with the findings of endogenous *O*-GlcNAc-modified Akt (**Fig. 1**), further strengthening the credibility of the Tyr mutation strategy in this scenario. Although well-validated in our study, caution should be taken when applying this approach to study the functions of *O*-GlcNAc modifications in a broader context.

Termination of a signal transduction is as essential as the activation for all signaling pathways, since the sustained hyperactivation will be detrimental to the normal physiological function [20]. In response to growth factor stimulation, Akt is activated and the phosphorylated Akt translocates to multiple subcellular organelles and interacts with over 130 substrates of Akt to transduce the signal to a variety of cellular processes [2]. Accordingly, the organisms have evolved multiple regulatory mechanisms to terminate the signal transduction of Akt, mainly including dephosphorylation by phosphatases [5], [13] and degradation by proteasome [15]. Here, we demonstrated *O*-GlcNAc modification as a new regulatory mechanism for attenuating Akt activity and functions, which advanced our understanding of the Akt pathway. Our studies uncovered an extensive and complex crosstalk between *O*-GlcNAcylations and phosphorylations of Akt. We employed the new antibody-based approach in combination with mass spectrometry and showed that Akt *O*-GlcNAcylations could affect almost all the phosphorylations of Akt, including Ser 126, Ser 129, Thr 308 and Ser 473. Moreover, the crosstalk between *O*-GlcNAcylations and phosphorylations of Akt was more complex than the “yin-yang hypothesis” [43], i.e. this interplay was not the simple binary model with reciprocal on or off status. On the one hand, *O*-GlcNAcylations of Akt could remarkably inhibit the phosphorylations of Akt, likely as the major role of Akt *O*-GlcNAcylations. On the other hand, Akt could simultaneously possess *O*-GlcNAcylations and phosphorylations, similar to IRS-1 *O*-GlcNAcylations [44]. Although it is still very difficult to elucidate the combinational effect of *O*-GlcNAcylations and phosphorylation using the existing technologies, the presence of *O*-GlcNAcylations will likely affect the biological functions of the adjacent phosphorylation. Together, our findings have shown that *O*-GlcNAcylation is a new regulator for Akt, with the broader roles in regulating Akt activity than previously speculated. The combinations of *O*-GlcNAcylations and phosphorylations will markedly increase molecular diversity of Akt [16]. These PTMs of Akt (either single PTM or PTMs in combination) will generate an array of binding surfaces for effector molecules and the downstream substrates to mediate the specific biological functions of Akt [7].

What is the functional contribution of the inhibitory *O*-GlcNAcylations to normal Akt signaling? It has been reported that Akt, PDK1 and OGT co-localize to the plasma membrane upon the activation of Akt signaling [20], where Akt is phosphorylated at Thr 308 by PDK1 and *O*-GlcNAcylated by OGT. In line with these previous reports, we identified the constitutive *O*-GlcNAcylations of Akt in MCF-7 breast cancer cells. Further, these *O*-GlcNAcylations under normal conditions inhibited the phosphorylations of Akt. The level of Akt activity at steady state should be the result of a balance between activation and inhibition events. The *O*-GlcNAcylation levels of Akt under normal physiological conditions may confer the optimal status for Akt signal transduction [20]. Therefore, these observations suggested that *O*-GlcNAc was necessary for normal Akt signaling. On the other hand, we utilized Tyr mutation to simulate the inhibition effect of *O*-GlcNAcylation. These mutants that represented an abnormal state of Akt *O*-GlcNAcylations, displayed the markedly reduced kinase activity, and in turn resulted in the significant loss of Akt normal biological functions. These findings indicated that the aberrant change of the balance between *O*-GlcNAc and *O*-phosphate of Akt would compromise the normal biological functions of Akt. Therefore, our studies open up a new avenue to understand Akt signal transduction through elucidation of the interaction between *O*-GlcNAcylations and phosphorylations of Akt.

In summary, we have developed a pair of new strategies to advance our understanding of the relationship between *O*-GlcNAc and *O*-phosphate of a given protein in complex biological systems. Our studies reveal an extensive and complex interplay between *O*-GlcNAcylations and phosphorylations of Akt and demonstrate *O*-GlcNAcylations as a new regulatory modification for Akt. These findings may have a direct implication in the regulatory mechanism of Akt signaling.

## Materials and Methods

### Reagents and Antibodies

Akt, phospho-Akt (Thr308 and Ser473), phospho-GSK3α/β (Ser21/9), PDK1, Na/K ATPase antibodies were obtained from Cell Signaling. *O*-GlcNAc antibody (CTD110.6) was from Covance. Anti-mouse-IgM agarose, dimethyl pimelimidate, were from Sigma. *O*-(2-acetamido-2-deoxy-D-glucopyranosylidene)amino-*N*-phenylcarbamate (PUGNAc) was obtained from Toronto Research Chemicals. Complete protease inhibitors cocktail and phosphatase inhibitors cocktail were obtained from Roche. Lipofectamine 2000 was obtained from Invitrogen. Anti-Flag agarose was obtained from Santa Cruz Biotechnology or Sigma.

### Cell Culture, Treatments and Transfection

MCF-7 and COS-7 cells were obtained from the American Type Culture Collection (Manassas, VA, USA) and maintained in DMEM media supplemented with 10% serum. Cells were grown to 80% confluence and then starved in the absence or the presence of 100 µM PUGNAc for 24 h, followed by stimulation with IGF-1 (50 ng/ml) for 15 min. A human AKT1 expression vector pcDNA3-Flag-HA-AKT1 (plasmid 9021) was from Addgene. Point mutants of Akt were generated with QuickChange site-directed mutagenesis technique. For cell transfection, MCF-7 and COS-7 cells were transfected using Lipofectamine 2000 according to the manufacture’s instruction.

### *O*-GlcNAc Immunoprecipitation and Immunoblotting

The *O*-GlcNAc antibody CTD110.6 was covalently immobilized to anti-IgM-agarose as described previously [45]. MCF-7 cells were lysed using RIPA lysis buffer containing protease/phosphatase inhibitors cocktail and PUGNAc. The cell lysate were incubated with CTD110.6 covalently crosslinked agarose. After extensively washed, the bound *O*-GlcNAcylated proteins were eluted by SDS-PAGE sampling buffer. Meanwhile, the supernatant were removed as the un-GlcNAcylated proteins. For western blotting assay, proteins were resolved by SDS-PAGE and transferred to nitrocellulose membrane. The corresponding strips of interest were blotted against the primary antibodies where indicated.

### Purification of Exogenous Akt and In-gel Digestion

MCF-7 cells transfected by wild-type AKT1 were treated by 100 µM PUGNAc for 24 h. Exogenous Akt was enriched by immunoprecipitation using anti-Flag agarose. The bound proteins were eluted by SDS-PAGE sampling buffer and further purified by SDS-PAGE. Akt bands were in-gel digested by trypsin or Glu-C at 37°C.

### Mass Spectrometry

LC-MS analysis was carried out on LTQ mass spectrometer (Thermo). Peptide mixture were separated on a C18 column. Mass spectrometric data were searched by SEQUST against human AKT1 protein sequence. The relevant searching parameters of mass range, intensity threshold, minimum ioncount, precursor ion mass tolerance and fragment ion mass tolerance were set as 500–5000, 500, 15, 3.0 and 1.0, respectively. Trypsin or Glu-C was designated as the protease, and up to two missed cleavages were allowed. Carbamidomethylation of Cys was as a fixed modification, and phosphorylation (80.0 Da) and *O*-GlcNAcylation (203.2 Da) of Ser/Thr, and oxidation of Met (16 Da) were allowed as variable modifications. All the MS/MS spectra corresponding to possible *O*-GlcNAcylated peptides were manually inspected according to the following criteria: 1) the fragment ions matched were clearly above baseline noise, 2) contain sequential series of b- or y-ions, 3) contain the characteristic fragment peaks of Pro-directed, Asp-directed and His-directed fragmentation, 4) *O*-GlcNAc neutral loss. The identified *O*-GlcNAcylated peptides were further evaluated according to the principle of sequence tagging [46].

### Molecular Modeling

The *O*-GlcNAc modifications on Thr 305 and Thr 312 sites were constructed on the X-ray crystal structure of AKT1 (PDB ID code: 3CQW), so as the Tyr 305 and Tyr 312 mutants. Molecular dynamics (MD) simulations were performed on the four systems plus wild type. MD simulations were carried out with constant temperature and pressure (NPT) and periodic boundary conditions. The AMBER Parm99 force field was applied for the proteins. The particle mesh Ewald method was used to calculate the long-range electrostatics interactions. The nonbonded cutoff was set to 10.0 Å, and the nonbonded pairs were updated every 25 steps. The SHAKE method was applied to constrain all covalent bonds involving hydrogen atoms. All simulations were coupled to 300 K at 1.0 atm of pressure (atm = 101.3 kPa).

### Immunofluorescence Microscopy

COS-7 cells were transfected by wild-type Akt and its mutants. After 36 h, cells were trypsinized and seeded on the coverslips and starved for 24 h, follows by IGF-1 stimulation for 15 min. The immunofluorescence assay for Akt was performed as described previously [20].

### Subcellular Fraction

COS-7 cells were transfected by wild-type Akt and its mutants. After 36 h, cells were starved for 24 h, followed by IGF-1 stimulation for 15 min. The membrane and cytosolic fractions from the stimulated cells were prepared as described previously [47].

### *In vitro* Akt and PDK1 Kinase Assays

MCF-7 cells were transfected with wild-type Akt and its mutants. For Akt kinase assay, cells were starved for 24 h, followed by IGF-1 stimulation for 15 min. Akt and its mutants were enriched from the transfected cells using anti-Flag agarose. *In* *vitro* Akt kinase assays were carried out according to the manufacture’s instruction (Cell Signaling). For PDK-1 kinase assay, Akt and its mutants were directly immunoprecipitated and incubated with PDK1 kinase according to the manufacture’s instruction (Cell Signaling).

### Cell Proliferation Assay

MCF-7 and COS-7 cells were transfected with wild-type Akt and its mutants. After 36 h, the transfected cells were trypsinized and 5×10^5^ cells were seeded in 24-well plate. At the culture time where indicated, cell were trypsinized and directly counted using automated cell counter. The data were expressed as the proliferation fold relative to the number of the initially seeded cells.

### Transwell Cell Migration Assay

COS-7 cells were transfected by wild-type Akt and its mutants. After 36 h, 1×10^5^cells were trypsinized and seeded into Transwell inserts. After 12 h, the upper side of the membrane was rubbed with cotton swap and the migrated cells in the basal side insert were fixed and stained with 0.1% crystal violet. The stained cells were extracted with a solution of 10% acetic acid, which absorbance was measured at 600 nm. The levels of cell migration were determined by comparison of the absorbance.

## Supporting Information

Figure S1 **Tyr substitutions simulate the inhibitory effect of** ***O*** **-GlcNAcylation of p53 and Snail1.** (**A**) S149Y p53 mutant shows the significantly reduced phosphorylation level of p53 at Thr 155. MCF-7 cells were transfected by the indicated plasmids.Wild type and S149Y mutants of p53 were immunoprecipitated with anti-Flag agarose and subjected to immunoblotting analysis of total p53 and the phosphorylation level of p53 at Thr 155. (**B**) S122Y Snail mutant shows the significantly reduced phosphorylation levels of Snail. MCF-7 cells were transfected by the indicated plasmids.Wild type and S122Y mutants of Snail were immunoprecipitated with anti-Flag agarose and were immunoblotted with total Snail and anti-phospho-serine antibody.
(TIF)
