# Supplementary figures and images for: Extensive Crosstalk between O-GlcNAcylation and Phosphorylation Regulates Akt Signaling
Source: PLoS One. 2012 May 22;7(5):e37427. doi: 10.1371/journal.pone.0037427 (PMC3358304; doi:10.1371/journal.pone.0037427)

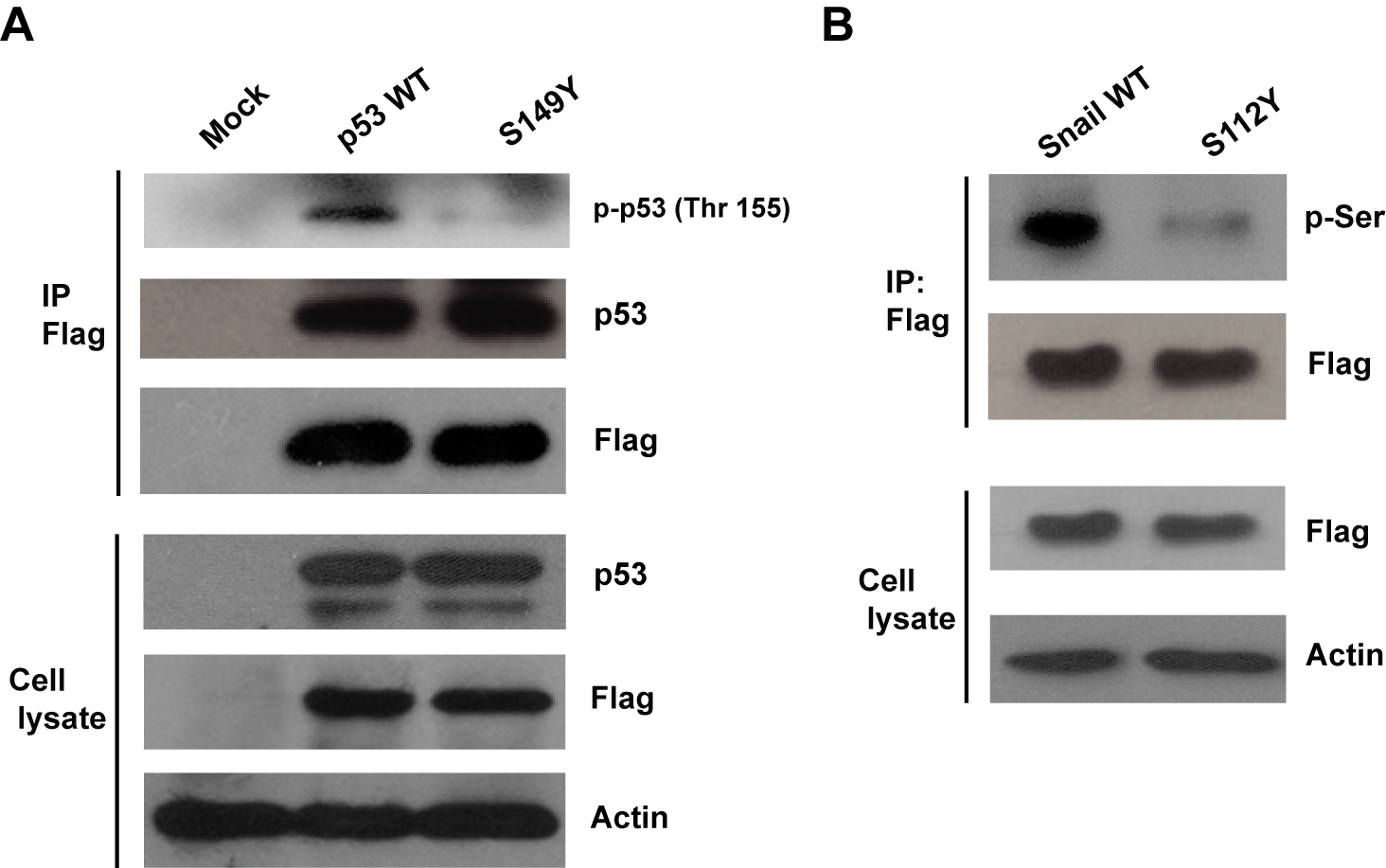

Supplement: Figure S1 — Tyr substitutions simulate the inhibitory effect of O -GlcNAcylation of p53 and Snail1. (A) S149Y p53 mutant shows the significantly reduced phosphorylation level of p53 at Thr 155. MCF-7 cells were transfected by the indicated plasmids.Wild type and S149Y mutants of p53 were immunoprecipitated with anti-Flag agarose and subjected to immunoblotting analysis of total p53 and the phosphorylation level of p53 at Thr 155. (B) S122Y Snail mutant shows the significantly reduced phosphorylation levels of Snail. MCF-7 cells were transfected by the indicated plasmids.Wild type and S122Y mutants of Snail were immunoprecipitated with anti-Flag agarose and were immunoblotted with total Snail and anti-phospho-serine antibody. (TIF) [file pone.0037427.s001.tif]
